# Adjuvant chemotherapy could not bring survival benefit to HR-positive, HER2-negative, pT1b-c/N0–1/M0 invasive lobular carcinoma of the breast: a propensity score matching study based on SEER database

**DOI:** 10.1186/s12885-020-6614-0

**Published:** 2020-02-21

**Authors:** Guangfu Hu, Guangxia Hu, Chengjiao Zhang, Xiaoyan Lin, Ming Shan, Yanmin Yu, Yongwei Lu, Ruijie Niu, Hui Ye, Cheng Wang, Cheng Xu

**Affiliations:** 1 Department of Breast Surgery, Huangpu Branch, Shanghai Ninth People’s Hospital, Affiliated to Shanghai Jiao Tong University School of Medicine, Shanghai, China; 2 Department of Pathology, Binzhong People’s Hospital, Affiliated to First Shandong Medical University, Binzhong, China; 3 Department of Psychological Measurement, Shanghai Mental Health Center, Shanghai Jiao Tong University School of Medicine, Shanghai, China; 4 Department of Breast Surgery, Yangpu Hospital, Tongji University School of Medicine, Shanghai, China

**Keywords:** Adjuvant chemotherapy, Breast cancer, Invasive lobular carcinoma, Hormone receptor-positive

## Abstract

**Background:**

The benefit of adjuvant chemotherapy in invasive lobular carcinoma (ILC) is still unclear. The objective of the current study was to elucidate the effectiveness of adjuvant chemotherapy in hormone receptor (HR)-positive, human epidermal growth factor receptor 2 (HER2)-negative, pT1b-c/N0–1/M0 ILC.

**Methods:**

Based on Surveillance, Epidemiology, and End-Results (SEER) database, we identified original 12,334 HR-positive, HER2-negative, pT1b-c/N0–1/M0 ILC patients, who were then divided into adjuvant chemotherapy group and control group. End-points were overall survival (OS) and breast cancer-specific mortality (BCSM). Aiming to minimize the selection bias of baseline characteristics, Propensity Score Matching (PSM) method was used.

**Results:**

In a total of 12,334 patients with HR-positive, HER2-negative, pT1b-c/N0–1/M0 ILC, 1785 patients (14.5%) were allocated into adjuvant chemotherapy group and 10,549 (85.5%) into control group. Used PSM, the 1785 patients in adjuvant chemotherapy group matched to the 1785 patients in control group. By Kaplan-Meier survival analyses, we observed no beneficial effect of adjuvant chemotherapy on OS in both original samples (*P* = 0.639) and matched samples (*P* = 0.962), however, ineffective or even contrary results of adjuvant chemotherapy on BCSM both in original samples *(P* = 0.001) and in matched samples (*P =* 0.002). In both original and matched multivariate Cox models, we observed ineffectiveness of adjuvant chemotherapy on OS (hazard ratio (HR) for overall survival = 0.82, 95% confidence interval (CI) [0.62–1.09]; *P* = 0.172 and HR = 0.90, 95%CI [0.65–1.26]; *P* = 0.553, respectively), unexpectedly promoting effect of adjuvant chemotherapy on BCSM (HR = 2.33, 95%CI [1.47–3.67]; *P* = 0.001 and HR = 2.41, 95%CI [1.32–4.39]; *P* = 0.004, respectively). Standard surgery was beneficial to the survival of patients. Lymph node metastasis was detrimental to survival and radiotherapy brought survival benefit in original samples, but two issues had unobvious effect in matched samples.

**Conclusion:**

In this study, adjuvant chemotherapy did not improve survival for patients with HR-positive, HER2-negative pT1b-c/N0–1/M0 ILC.

## Background

Invasive lobular carcinoma (ILC) is the most common ‘special’ morphological subtype of breast cancer and presents with a distinctive clinical behavior compared with invasive ductal carcinoma (IDC) (no special type) [1, 2]. Classical ILC is characterized by monotonous small, uniform, discohesive cells that infiltrate the stroma in a single-file pattern [2]. This distinctive feature of classical ILC results from the E-cadherin loss on tumor cell membranes [3, 4]. Importantly, loss of E-cadherin not only results in a dysfunctional E-cadherin-catenin complex with consequences on cell-cell adhesion but also the different inter-cellular and intracellular signaling pathways [5]. Therefore, ILC should be considered to a distinct entity different from IDC [6, 7].

Generally, ILC displays features associated with hormone receptor (HR)-positive, human epidermal growth factor receptor 2 (HER2)-negative, being low grade and a good prognosis [8]. It is generally admitted that ILC, especially HR-positive, HER-2-negative ILC, is endocrine responsive, and responds poorly to chemotherapy [9, 10]. So for it is not yet settled in published clinical studies that whether adjuvant chemotherapy is effective for relatively early stage patients with HR-positive, HER2-negative ILC.

In 2011, based on a Dutch regional cohort of 498 ILC patients, Truin et al. [11] reported that overall survival (OS) was not statistically different in HR-positive ILC patients treated with adjuvant endocrine therapy and chemotherapy compared to adjuvant endocrine therapy alone (5-year OS 85.2% vs 82.8%, *P* = 0.68). In 2012, using the data from the Netherlands Cancer Registry (NCR) of 3685 ILC patients, Truin et al. [12] also reported that adjuvant chemotherapy seems to confer no additional beneficial effects in postmenopausal patients with pure or mixed type ILC (10-year OS 66% vs 68%, *P* = 0.45). In 2017, identifying 4638 ILC from California Cancer Registry (CCR), Marmor et al. [13] determined a similar result that patients with estrogen receptor (ER)-positive, HER2-negative, stage I/II ILC who received adjuvant endocrine therapy did not benefit from the addition of adjuvant chemotherapy. However, using 2318 ILC data source from 15 academic French cancer centers between 1990 and 2014, Nonneville et al. [14] recently reported that the significant disease-free survival (DFS) and OS benefits from adjuvant chemotherapy could be derived in high-risk ILC patients, but not in low-risk ILC patients.

In a dilemma, how should we make a treatment choice for HR-positive, HER2-negative, pT1–2/N0–1/M0 ILC, especially, the pT1b-c/N0–1/M0 ILC? The Surveillance, Epidemiology, and End-Results (SEER) database is publicly available for studies of cancer-based epidemiology and TNM staging of breast cancer and other cancers, which covers approximately 28% of the US population [7, 15]. Using SEER database, the aims of our study were to confirm whether the adjuvant chemotherapy could bring survival benefit to patients with HR-positive, HER2-negative, pT1b-c/N0–1/M0 ILC. To our knowledge, this is the first and the largest, population-based study presenting evidence of effect of adjuvant chemotherapy in patients with ILC used SEER database. Above all, our findings have a direct and meaningful translation to the clinic, allowing us to avoid excessive adjuvant chemotherapy for patients with HR-positive, HER2-negative, pT1b-c/N0–1/M0 ILC.

## Methods

### Data source and study design

**Fig. 1:**
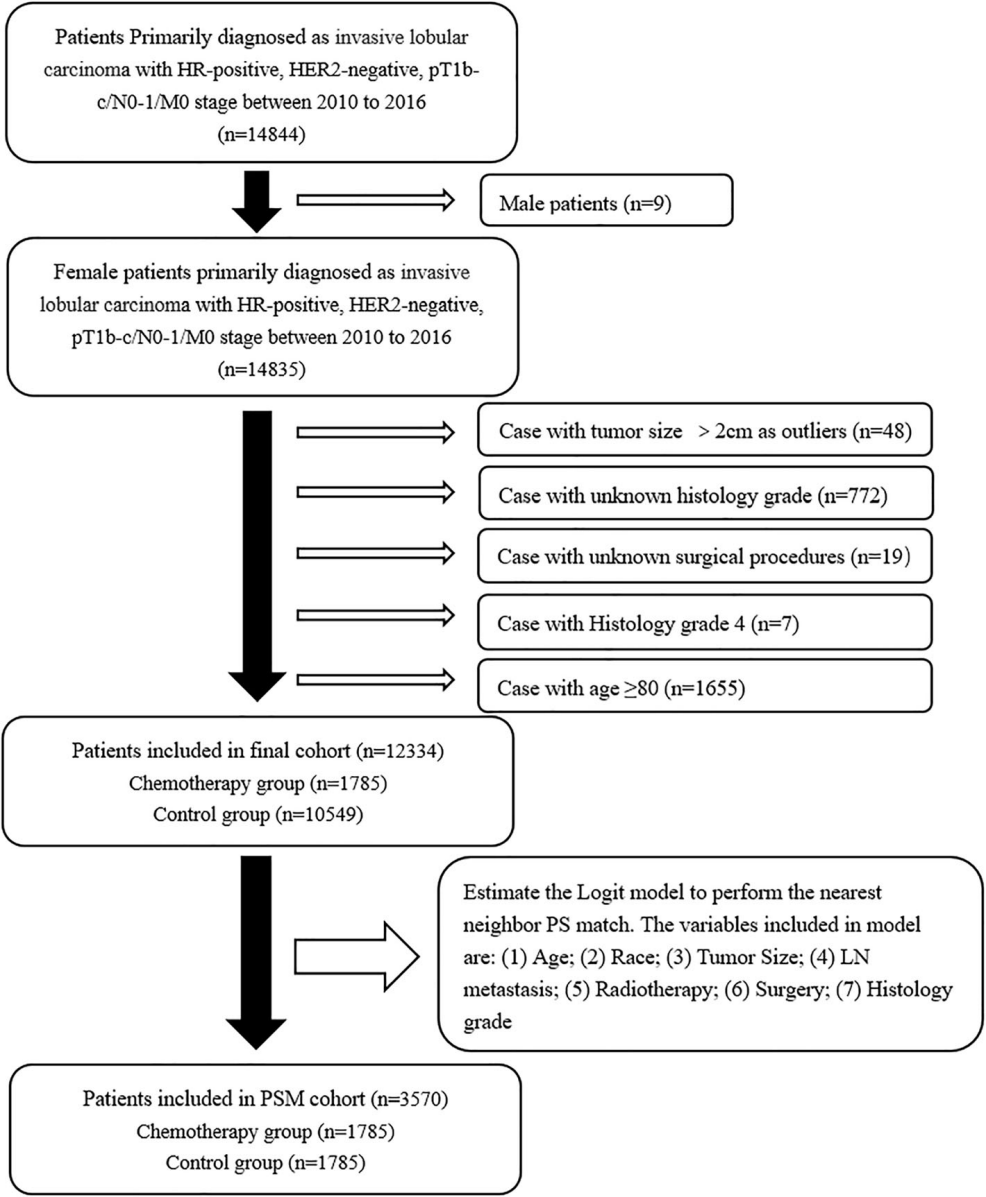
Flow diagram of patient selection and study development

The SEER program is a national database and primary source of cancer statistics that is currently maintained by the National Cancer Institute. We have got permission to access the database and reproduce individual data in SEER*Stat Database: Incidence - SEER 18 Regs Custom Data (with additional treatment fields), Nov 2018 Sub (1975–2016 varying) - Linked To County Attributes - Total U.S., 1969–2017 Counties, National Cancer Institute, DCCPS, Surveillance Research Program, released April 2019, based on the November 2018 submission. We obtained patients diagnosed with ILC of pT1b-c/N0–1/M0 according to Site Recode classification and AJCC 7th ed. stage system. The collected patients were diagnosed from 2010 to 2016, because breast cancer subtype was available in SEER database since 2010. We retrieved 14,844 record of HR-positive, HER2-negative, pT1b-c/N0–1/M0 ILC (Supplementary Table 1). After omitting censored data and excluding patients older than 80 years old, a total of 12,334 patients were enrolled in our study (Fig.1).

### Statistical analysis

The differences of demographic and clinic-pathological features between chemotherapy group and control group were analyzed by chi-square and Wilcoxon ranksum test. Propensity Score Matching (PSM) method (Match Ratio 1:1; Logit model; the nearest neighbor matching approach) was used to eliminate clinic-pathological mixed bias in two groups (Supplementary dofile). Overall survival (OS) was defined as the time from admission to the date of death from any cause. Breast cancer-specific mortality (BCSM) was defined as the period from the operative date to death of breast cancer. The OS curves and BCSM curves of each group were estimated by Kaplan-Meier survival analyses, and the curves were analyzed by the log-rank test. In the multivariate analysis, a COX’s Proportional Hazard Model was employed to estimate whether a factor was a significant independent prognostic factor of survival. All statistical tests were two-sided, *P* values less than 0.05 were considered as statistically significant. The statistical analyses were performed using STATA version 15.1 for windows (StataCorp LLC).

## Results

### Characteristics of the original patients

After omitting censored data and excluding patients older than 80 years old, an original of 12,334 patients with HR-positive, Her-2-negative, pT1b-c/N0–1/M0 ILC were enrolled in our study (Supplementary Table 2). In total patients, 1785 patients (14.5%) received the adjuvant chemotherapy (chemotherapy group) and 10,549 patients (85.5%) not (control group). Compared with patients of control group, patients of chemotherapy group presented significantly more adverse prognostic features, such as young age (58.49% vs. 32.83% age < 60, *P* < 0.05), larger tumor size (79.05% vs. 67.25% T1c, *P* = 0.001), high grade (75.91% vs. 65.7% grades II&III, *P* < 0.05), more lymph node involvement (49.86% vs. 11.76% pN1, *P* = 0.001). Patients of chemotherapy group underwent more mastectomy (50.81% vs. 34.08%, *P* < 0.05), but less radiotherapy (50.53% vs. 54.74%, *P* < 0.05). The comparisons of characteristics between two groups were shown in Table 1.

**Table 1:** Comparisons of characteristics between chemotherapy group and control group in original 12,334 HR-positive, Her-2-negative, pT1b-c/N0–1/M0 ILC patients

		Control (*n* = 10,549)	Chemotherapy (*n* = 1785)	Statistical value	*P*	bias	t-test for bias	*P*
Age (years)	< 40	59 (0.56%)	47 (2.63%)	χ2 = 576.457	0.001	−62.1	−24.37	0.001
	40–49	1088 (10.31%)	392 (21.96%)					
	50–59	2316 (21.95%)	605 (33.89%)					
	60–69	3896 (36.93%)	557 (31.20%)					
	70–79	3190 (30.24%)	184 (10.31%)					
Race	Black	868 (8.23%)	174 (9.75%)	χ2 = 4.627	0.099	−4.6	−1.81	0.071
	White	8966 (84.99%)	1495 (83.75%)					
	Other	715 (6.78%))	116 (6.50%))					
Tumor	Ib	3455 (32.75%)	374 (20.95%)	χ2 = 99.294	0.001	26.9	10.00	0.001
	Ic	7094 (67.25%)	1411 (79.05%)					
LN	N0	9308 (88.24%)	895 (50.14%)	χ2 = 1.6e+ 03	0.001	90.6	42.10	0.001
	N1	1241 (11.76%)	890 (49.86%)					
Grade	I	3618 (34.30%)	430 (24.09%)	z = −10.373	0.001	27.6	10.87	0.001
	II	6472 (61.35%)	1188 (66.55%)					
	III	459 (4.35%)	167 (9.36%)					
Radiotherapy	No	4774 (45.26%)	883 (49.47%)	χ2 = 10.910	0.001	−8.4	−3.30	0.001
	Yes	5775 (54.74%)	902 (50.53%)					
Surgery	No surgery	177 (1.68%)	36 (2.02%)	χ2 = 190.161	0.001	31.5	12.61	0.001
	BCS	6777 (64.24%)	842 (47.17%)					
	Mastectomy	3595 (34.08%)	907 (50.81%)					

### PSM method to minimize the selection bias of baseline characteristics

**Fig. 2:**
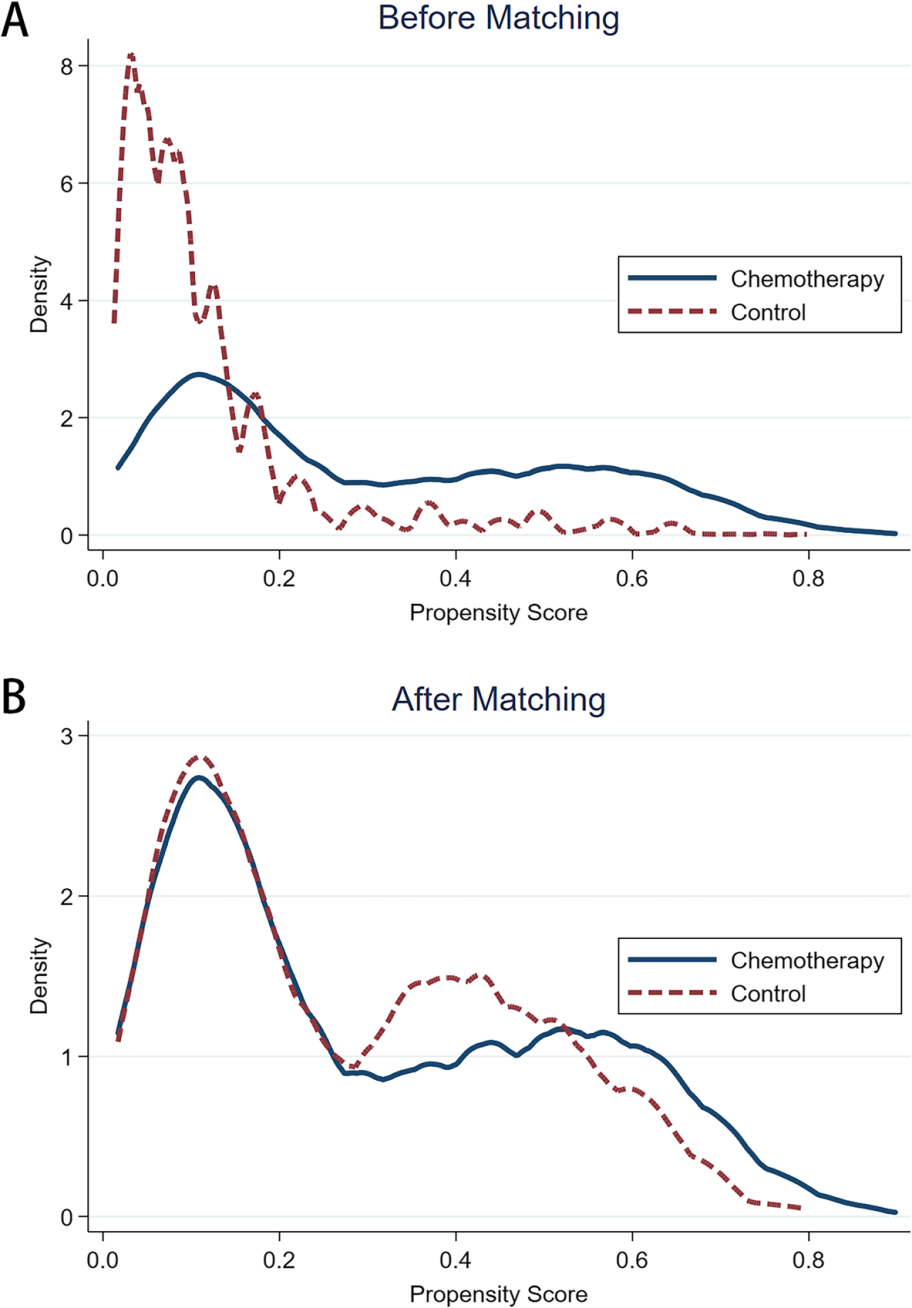
**a** Kernel Density of the chemotherapy and control groups before PS matching; **b** Kernel Density of the chemotherapy and control groups after PS matching

In order to study the effect of chemotherapy on survival by equilibrium, we employed PSM method (Match Ratio 1:1) to minimize the selection bias of demographic and clinic-pathological characteristics between the two groups. The kernel density functions of the chemotherapy group and the control group, based on pre-matching showed that the characteristics of the variables in the two groups had remarkable bias (Fig.2a). After matching, as shown in Fig.2b, the kernel density functions of the chemotherapy group and the control group (1785 patients from original control group) were a lot closer, indicating that the clinic-pathological characteristics in chemotherapy group and the control group are similar (Supplementary Table 3).

### Characteristics of the matched patients

The matched results showed that the bias between chemotherapy group and the control group had unobvious statistically significant (bias< ± 10, *P*>0.05). In matched samples, apart from age (*P* = 0.001), almost all of the demographic and clinic-pathological characteristics were similarly distributed between chemotherapy group and control group (*P*>0.05) (Table 2).

**Table 2:** Comparisons of characteristics between chemotherapy group and control group 2 in matched 3570 HR-positive, Her-2-negative, pT1b-c/N0–1/M0 ILC patients

		Control (*n* = 1785)	Chemotherapy (*n* = 1785)	Statistical value	*P*	bias	t-test for bias	*P*
Age (years)	< 40	37 (2.07%)	47 (2.63%)	χ2 = 15.831	0.003	−11.6	−3.48	0.001
	40–49	319 (17.87%)	392 (21.96%)					
	50–59	588 (32.94%)	605 (33.89%)					
	60–69	646 (36.19%)	557 (31.20%)					
	70–79	195 (10.92%)	184 (10.31%)					
Race	Black	162 (9.08%)	174 (9.75%)	χ2 = 0.170	0.919	−3.7	−1.08	0.280
	White	1493 (83.64%)	1495 (83.75%)					
	Other	130 (7.28%)	116 (6.50%)					
Tumor	Ib	389 (21.79%)	374 (20.95%)	χ2 = 0.375	0.540	1.9	0.61	0.540
	Ic	1396 (78.21%)	1411 (79.05%)					
LN	N0	907 (50.81%)	895 (50.14%)	χ2 = 0.161	0.688	1.6	0.40	0.688
	N1	878 (49.19%)	890 (49.86%)					
Grade	I	450 (25.21%)	430 (24.09%)	z = −0.762	0.446	2.5	0.75	0.456
	II	1173 (65.71%)	1188 (66.55%)					
	III	162 (9.08%)	167 (9.36%)					
Radiotherapy	No	938 (52.55%)	883 (49.47%)	χ2 = 3.391	0.066	6.2	1.84	0.066
	Yes	847 (47.45%)	902 (50.53%)					
Surgery	No surgery	34 (1.90%)	36 (2.02%)	χ2 = 3.392	0.183	5.5	1.59	0.112
	BCS	897 (50.25%)	842 (47.17%)					
	Mastectomy	854 (47.84%)	907 (50.81%)					

### OS and BCSM analysis before or after matching

**Fig.3:**
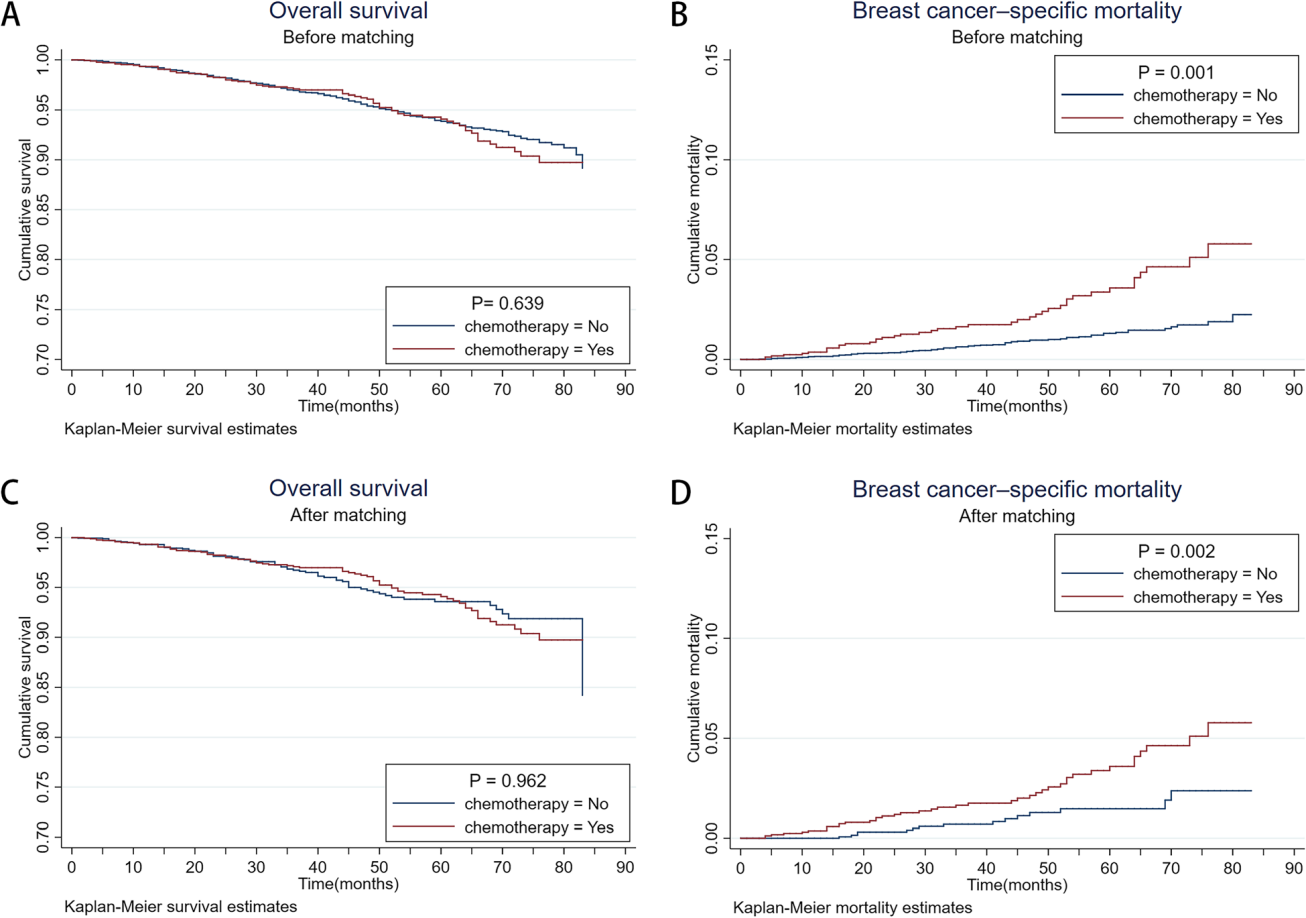
**a** Kaplan–Meier analyses of the effect chemotherapy on OS in original samples (*P* = 0.639, log-rank test); **b** Kaplan–Meier analyses of the effect chemotherapy on BCSM in original samples (*P* = 0.001, log-rank test); **c** Kaplan–Meier analyses of the effect chemotherapy on OS in matched samples (*P* = 0.962, log-rank test); **d** Kaplan–Meier analyses of the effect chemotherapy on BCSM in matched samples (*P* = 0.002, log-rank test)

In the original 12,334 patients with HR-positive, Her-2-negative, T1b-c/N0–1/M0 ILC were followed-up for a median of 42 months (range of 1 to 83 months). By the end of follow-up period, 74 of 1785 patients (chemotherapy group) had died, 41 patients died of breast cancer, with the corresponding, 361 of 10,549 patients (control group) had died, 76 patients due to recurrence and metastasis of breast cancer. Thus before matching, the OS of the chemotherapy group had no obviously difference than that of the control group (*P* = 0.639, log-rank test) (Fig.3a). However, the BCSM of the chemotherapy group was higher than that of the control group, which reach distinct levels of statistical significance (*P* = 0.001, log-rank test) (Fig.3b).

After matching, 66 of 1785 patients in the control group had dead, 15 of whom owing to the breast cancer. The OS curve of chemotherapy group and control group interwove with each other (*P* = 0.962, log-rank test) (Fig.3c). Unexpectedly, the BCSM of the chemotherapy group was still statistical significantly higher than that of the control group (*P =* 0.002, log-rank test) (Fig.3d). Accordingly, adjuvant chemotherapy was likely on the contrary to induced more breast cancer mortality.

### The original and the matched multivariate Cox proportional hazards models for OS and BCSM

**Fig.4:**
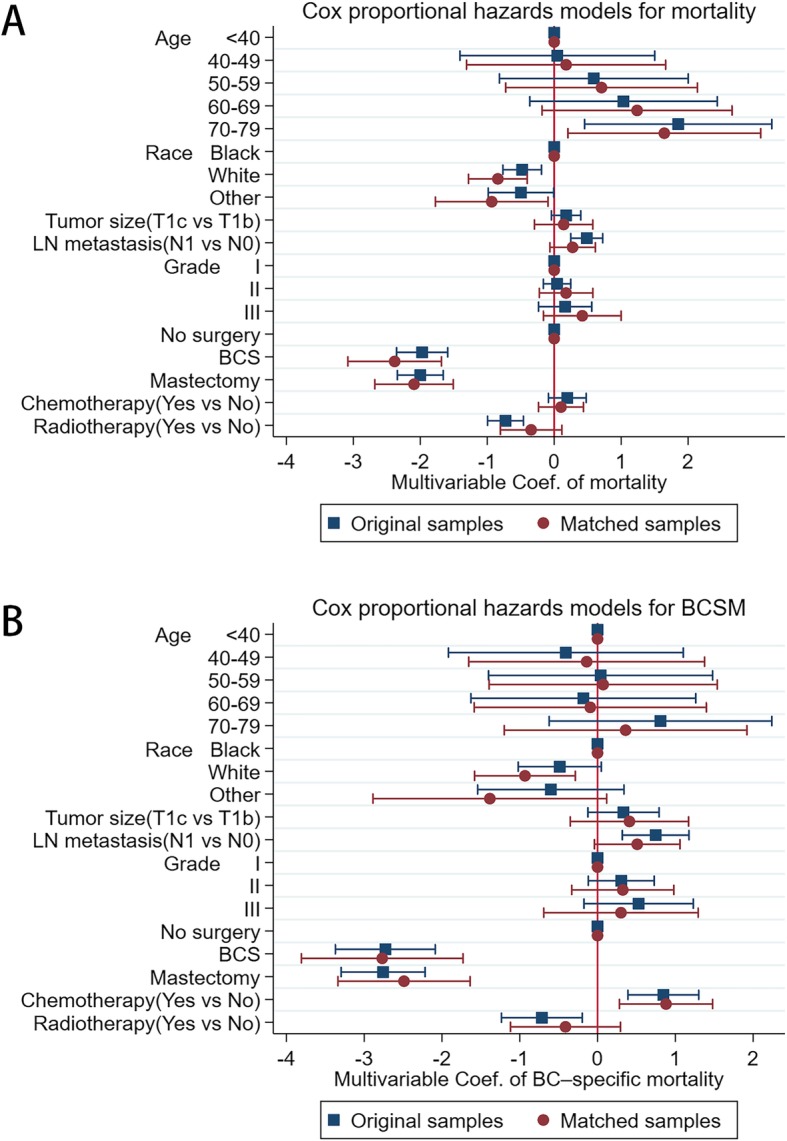
**a** Cox proportional hazards models for overall mortality before and after matching; **b** Cox proportional hazards models for BCSM before and after matching

To adjust potential modifier effects to adjuvant chemotherapy, both the original and the propensity score matched multivariate Cox proportional hazards models were fitted for overall survival and BCSM. As shown in Fig.4a and Table 3, adjuvant chemotherapy did not bring overall survival benefit in both original and matched Cox models (HR = 0.82, 95%CI [0.62–1.09]; *P* = 0.172 and HR = 0.90, 95%CI [0.65–1.26]; *P* = 0.553, respectively). However, as shown in Fig.4b and Table 3, adjuvant chemotherapy unexpectedly increased the risk of BCSM in both original and matched Cox models (HR = 2.33, 95%CI [1.47–3.67]; *P* = 0.001 and HR = 2.41, 95%CI [1.32–4.39]; *P* = 0.004, respectively). Additionally, in both original and matched Cox models, standard surgery was negatively correlated with the risk of BCSM and improved overall survival (all coefficients < 0, *P* < 0.05) shown in Fig.4a, Fig.4b and Table 3. Advanced age was a pernicious factor for overall survival. Lymph node metastasis was positively related to both poor overall survival and risk of BCSM in original Cox models (all coefficients > 0, *P* < 0.05), however, they were no longer significant for overall survival and BCSM in matched Cox models shown in Fig.4a, Fig.4b and Table 3. The effect of radiotherapy was just opposite to lymph node metastasis. In original samples, the prognosis of white race patients is better than that of black race. This trend gets still more obvious in matched samples (HR for OS > 1 and HR for BCSM < 1, *P* < 0.05). High histological grade had no implicit relation with the risk of overall survival and BCSM in both original and matched samples (all coefficients > 0, but *P* > 0.05).

**Table 3:** Multivariate Analyses of OS and BCSM in original samples and matched samples

Variable	original samples							matched samples					
		HR for	95%CI	P	HR for	95%CI	P	HR for	95%CI	P	HR for	95%CI	P
		OS			BCSM			OS			BCSM		
Age (years)	< 40	Reference			Reference			Reference			Reference		
	40–49	0.95	0.22–4.08	0.949	0.66	0.14–3.01	0.597	0.84	0.19–3.70	0.814	0.86	0.19–3.96	0.857
	50–59	0.55	0.14–2.27	0.410	1.04	0.24–4.39	0.957	0.49	0.12–2.06	0.333	1.07	0.24–4.65	0.923
	60–69	0.36	0.09–1.44	0.147	0.83	0.19–3.53	0.805	0.29	0.07–1.20	0.087	0.91	0.20–4.05	0.903
	70–79	0.16	0.04–0.63	0.009	2.24	0.53–9.39	0.268	0.19	0.05–0.81	0.025	1.43	0.30–6.82	0.650
Race	Black	Reference			Reference			Reference			Reference		
	White	1.61	1.21–2.15	0.001	0.61	0.36–1.04	0.075	2.32	1.50–3.60	0.001	0.39	0.20–0.75	0.005
	Others	1.64	1.01–2.68	0.047	0.54	0.21–1.40	0.211	2.54	1.10–5.90	0.030	0.25	0.05–1.12	0.071
Tumor	(Ic vs Ib)	0.84	0.67–1.04	0.111	1.39	0.88–2.20	0.153	0.87	0.56–1.34	0.524	1.50	0.70–3.22	0.290
LN	(N1 vs N0)	0.61	0.48–0.78	0.001	2.11	1.37–3.24	0.001	0.76	0.54–1.07	0.112	1.66	0.96–2.88	0.069
Grade	I	Reference			Reference			Reference			Reference		
	II	0.96	0.78–1.18	0.679	1.35	0.88–2.07	0.158	0.84	0.56–1.25	0.384	1.38	0.71–2.66	0.332
	III	0.85	0.57–1.26	0.418	1.69	0.84–3.42	0.141	0.66	0.37–1.17	0.156	1.35	0.50–3.64	0.552
Surgery	No surgery	Reference			Reference			Reference			Reference		
	BCS	7.19	4.91–10.53	0.001	0.06	0.03–0.12	0.001	10.84	5.39–21.80	0.001	0.06	0.02–0.17	0.001
	Mastectomy	7.39	5.24–10.41	0.001	0.06	0.03–0.10	0.001	8.11	4.51–14.58	0.001	0.08	0.03–0.19	0.001
Chemotherapy	(Yes vs No)	0.82	0.62–1.09	0.172	2.33	1.47–3.67	0.001	0.90	0.65–1.26	0.553	2.41	1.32–4.39	0.004
Radiotherapy	(Yes vs No)	2.07	1.58–2.70	0.001	0.48	0.29–0.82	0.007	1.41	0.89–2.23	0.142	0.66	0.32–1.34	0.253

## Discussion

As well as HR and HER2 status, some studies have indicated that the histological subtype of the breast cancer also plays an important role in predicting the response to adjuvant chemotherapy and/or neoadjuvant chemotherapy (NAC) [16–19]. In 2005, Cristofanilli et al. [16, 17] reported that ILC is characterized by lower pathologic complete response (pCR) rates to NAC but better long-term outcomes compared to IDC. In 2007, Katz et al. [18] reviewed randomized trials of NAC and noted that the pCR rate was 1.7% in ILC and 11.6% in IDC (no special type). In 2010, in the era of tailored therapy for individual patients, Purushotham et al. [19] documented that we would no longer routinely recommend NAC in patients with ER-positive, HER2-negative, classical type ILC.

However, though it is generally admitted that ILC, especially HR-positive, HER2-negative ILC, responds poorly to chemotherapy, currently available data do not unanimously support these assumptions. In 2012, Lips et al. [9] reported a similar pCR rate in both ER-positive, HER2-negative IDC and ER-positive, HER2-negative ILC patients (4.2 and 4.3%, respectively). In 2014, Guiu et al. [20] reported that in multivariate analysis, histology of ILC was not an independent negative predictive factor of pCR in seven [21–27] of nine studies [21–29].

Thus, we could not draw a conclusion that ILC or even HR-positive, HER2-negativeis ILC is an independent predictor of poor response to adjuvant chemotherapy and/or NAC. In fact, minority of past and current studies take lobular histology into account in pretreatment stratification or subgroup analysis. Consequently, findings of these studies limit our ability to indicate whether patients with IDC or ILC should be managed with similar or different treatments. At present, the National Comprehensive Cancer Network (NCCN) and the St Gallen International Expert Consensus guidelines for systemic therapy decisions are almostly derived from studies based on IDC. Neither of these two guidelines consider histologic subtype as a factor for determining systemic therapy decisions. Making systemic therapy decisions for patients with ILC is thus challenging for the oncology community. It is unlikely that a future randomized clinical trial (RCT) concerning this subject will be accomplished. There is lack of stronger evidence support, this may be why our guidelines still do not distinguish ILC from IDC for treatment allocation or classification therapy.

In this study by using SEER database, we firstly compared the cohort characteristics between the included HR-positive, HER2-negative, pT1b-c/N0–1/M0 ILC patients with and without adjuvant chemotherapy, in both original and propensity score matched sample, respectively. Secondly, OS and BCSM analyses between chemotherapy and control groups were made, before or after PSM, respectively. Thirdly, to adjust the potential confounding factors to chemotherapy, the multivariate Cox regression analyses were performed for overall survival and BCSM, in both original and matched sample, respectively. Our data demonstrate that patients with HR-positive, HER2-negative, pT1b-c/N0–1/M0 ILC could not derive survival benefit from the adjuvant chemotherapy shown in Fig.2, Fig.3 and Table 3, neither for OS nor for BCSM. In both original and propensity score matched sample, ILC patients with adjuvant endocrine therapy and chemotherapy had a worse BCSM than ILC patients with adjuvant endocrine therapy alone. This finding is almost certainly secondary to selection bias and not cause and effect of adjuvant chemotherapy.

Histological grading is an important part of breast cancer classification, and is performed using the Nottingham histological grading system. However, it has been controversial as to the relevance of this system for ILC, since tubule formation is rare (except in the tubulo-lobular variant) [30]. With limited nuclear pleomorphism and sparse mitotic count, ILC (including variants) is often characterized by lower histologic grade compared to IDC [31]. In both our original and matched samples, almost or more than ninety percentages of ILC were histologic grade 1–2 (Table 1 and Table 2). Consequently, a therapeutic dilemma can occur in the event of the relative resistance of ILC to conventional chemotherapeutic agents [32, 33]. Moreover, lack of E-cadherin protein expression in ILC is distinctive from IDC [34]. It has been hypothesized that the lack of chemosensitivity of ILC is explained by the inactivation of E-cadherin in ILC. Loss of E-cadherin protein is thought to increase of epithelial to mesenchymal transition (EMT), which in turn become more resistant to chemotherapy [35]. Accordingly, lower histologic grade and deficiency of E-cadherin of ILC both supported our results.

It has been demonstrated that ILC and IDC have distinct genomic, transcriptomic and expression profiles [36]. Recent major advances in genome-wide transcription analyses, comparative genomic hybridization (CGH) and genomic tests further acknowledged the natural history and also the heterogeneity of ILC [37]. It has been suggested that the genomic signatures could be used to assist systemic therapy decisions for patients with ILC, and especially the decision of adding chemotherapy to hormonal therapy [38]. For instance, mutations in exon 9 of the PIK3CA gene have previously been reported more frequent in ILC than in IDC [39–41]. These mutations increase kinase activity, confer increased resistance to paclitaxel and are associated with metastatic capability [42, 43]. Intriguingly, loss of E-cadherin of ILC has been also associated with many genetic and molecular alterations including the inactivation of the CDH1 gene at 16q22 by mutation, loss of heterozygosity, or CDH1 promoter methylation, which finally lead to the poor response to cytotoxic chemotherapy [3, 4, 44].

Oncotype Dx Breast Cancer Assay is a 21-gene assay used in estrogen receptor (ER)-positive breast cancer to predict benefit from chemotherapy [45, 46]. In 2015, Conlon et al. [47] reported that Oncotype Dx recurrence score (RS) currently plays a clinically useful role in the management of ILC, which may prevent the over-treatment of adjuvant chemotherapy. In 2017, Kizy et al. [48] reported that patients with ER-positive ILC, 8% were in the high-risk and 72% were in the intermediate-risk groups as per the trial assigning individualized options for treatment (TAILORx) RS cutoffs. Adjuvant chemotherapy did not seem to confer a survival benefit for either the intermediate- or the high-risk cohorts [48].

Some limitations of our study have to be considered, thus we ought to be caution about our results. Our SEER database does not include information regarding the ILC and its variants, the loss of E-cadherin, the exact ER and PR and Ki67 expression, the 21-gene assay, the administration of chemotherapy and endocrine therapy, ect. Additionally, we should exclude all cases where breast cancer has only been reported by death certificate or autopsy. Thirdly, there is indeed an important deficiency is that the chemotherapy record in SEER database is classified as “No/Unknown” and “Yes”. Although we obtained data of 1785 patients with definite chemotherapy from SEER database, we don’t know whether the patients recorded as “No/Unknown” actually received chemotherapy. All these confounding factors may have affected our results. For example, the most recent 2012 WHO classification of breast cancer distinguishes the ILC and its variants: classic, solid, alveolar, pleomorphic, tubulo-lobular, and mixed variant [1]. Lack of E-cadherin is observed in all histological ILC variants, except for tubulo-lobular variant (tubulo-lobular carcinoma, TLC). Pleomorphic variant (pleomorphic invasive lobular carcinoma, PILC) shares many additional genomic changes with classic ILC such as TP53 stabilization, amplifications of MYC, MDM2, HER2/TOP2A and 20q13 [49].

Our study is subject to some methodologic limitations too, which will lead to inevitable bias. The present study is a retrospective cohort study, however, not a RCT. The patient demographics and tumor characteristics are not totally consistent between the included ILC patients with and without adjuvant chemotherapy, even though after PSM analysis. Furthermore, the PSM analysis is also limited by the lack of adjustment for the cointervention of surgery therapy or radiation therapy, which demotivates our study due to reduce the sample sizes or event rates.

Nevertheless, until now, it is not clear whether there is a difference ineffectiveness between chemotherapy regimens administered to patients with ILC. Therefore, we suggest that further research on the type of chemotherapy administered to patients with ILC should be carried out. Moreover, evaluation of the response of ILC patients to endocrine therapy is an emerging direction of clinical breast cancer research [50]. It was reported that the magnitude of benefit of adjuvant letrozole is greater for patients diagnosed with ILC compared to those with IDC [51]. In fact, it may be time for the oncologists to consider a prospective RCT to evaluate the role of NAC versus neoadjuvant endocrine therapy in ILC patients [18]. Additionally, whether CDK4/6 inhibitor is more effective for HR-positive, HER2-negative ILC than for HR-positive, HER2-negative IDC is worth to study. Finally, we advise to the oncologists that ILC and its variants should be studied, with further efforts made to develop more individualized treatment for them and to identify potential mechanisms of their biological inferiority and superiority, respectively [52, 53].

## Conclusion

Adjuvant chemotherapy could not improve survival for patients with HR-positive, HER2-negative pT1b-c/N0–1/M0 ILC.

## Supplementary information

**Supplementary Table 1.** 14,844 initial samples - 14,844 initial record of HR-positive, HER2-negative, pT1b-c/N0–1/M0 ILC retrieved from SEER database.

**Supplementary Table 2.** 12,334 original samples - 12,334 record of HR-positive, HER2-negative, pT1b-c/N0–1/M0 ILC enrolled in the study.

**Supplementary Table 3.** 3570 matched samples - 3570 record of HR-positive, HER2-negative, pT1b-c/N0–1/M0 ILC (including 1785 matched non-chemotherapy patients and 1785 chemotherapy patients).

**Additional file 4.** Supplementary dofile: dofile for Stata

## Data Availability

All data generated or analyzed during this study are included in this published article [and its sementary information files].

## Abbreviations

BCSM: Breast cancer-specific mortality
CGH: Comparative genomic hybridization
CI: Confidence interval
DFS: Disease-free survival
EMT: Epithelial to mesenchymal transition
ER: Estrogen receptor
HER2: Human epidermal growth factor receptor 2
HR: Hazard ratio
HR: Hormone receptor
IDC: Invasive duct carcinoma of no special type
ILC: Invasive lobular carcinoma
NAC: neoadjuvant chemotherapy
NCCN: National Comprehensive Cancer Network
OS: Overall survival
pCR: Pathologic complete response
PSM: Propensity score matching
RCT: randomized clinical trial
RS: Recurrence score
SEER: Surveillance, Epidemiology, and End-Results database
